# Risk of extrapyramidal symptoms associated with antipsychotic drugs in children and adolescents: a systematic review and meta-analysis of double-blind randomised placebo-controlled trials

**DOI:** 10.21203/rs.3.rs-9601341/v1

**Published:** 2026-05-25

**Authors:** Joaquín Galvañ, Giacomo Distefano, Iván Echeverria, Clàudia Aymerich, Renzo Abregú-Crespo, Marién Martín Argila Lorente, Nadia Cruz, Laura López-Larriba, Dolores Moreno, Mara Parellada, Celso Arango, Gonzalo Salazar Pablo, Covadonga M. Díaz-Caneja

**Affiliations:** Hospital General Universitario Gregorio Marañón, Universidad Complutense; Ospedale Maggiore CA Pizzardi, AUSL; Consorcio Hospitalario Provincial de Castellón; King’s College London; Hospital General Universitario Gregorio Marañón, Universidad Complutense; Hospital Universitario Dr. Rodríguez Lafora; Hospital General Universitario Gregorio Marañón, Universidad Complutense; Hospital General Universitario Gregorio Marañón, Universidad Complutense; Hospital General Universitario Gregorio Marañón, Universidad Complutense; Hospital General Universitario Gregorio Marañón, Universidad Complutense; Hospital Universitario La Paz, Universidad Autónoma de Madrid; King’s College London; Hospital General Universitario Gregorio Marañón, Universidad Complutense

## Abstract

**Background:**

Antipsychotic drugs are increasingly prescribed to children and adolescents (C&A) across a range of neuropsychiatric and neurodevelopmental disorders. Extrapyramidal symptoms (EPS) are well-established adverse effects of antipsychotics in adults, yet evidence in paediatric populations remains limited. This study aimed to quantify the risk of antipsychotic-related EPS in C&A and to explore potential moderating factors.

**Methods:**

This PRISMA-compliant systematic review and meta-analysis (PROSPERO; CRD42024564227) searched MEDLINE, Embase, and Web of Science from inception until the 6th of January 2026, for double-blind randomised controlled trials (RCTs) comparing antipsychotics with placebo in individuals aged < 18 years. EPS were assessed through either adverse event reporting or predefined thresholds on validated clinical rating scales and harmonised into a dichotomous outcome (presence vs. absence). Data were extracted by three pairs of independent researchers. Random-effects meta-analyses were conducted to estimate pooled risk ratios (RRs), alongside meta-regression and subgroup analyses examining potential moderators. Risk of bias was assessed using Cochrane Rob2.

**Results:**

Thirty-seven RCTs including 5,128 participants (3,141 receiving antipsychotics, 1,987 receiving placebo) were included. Exposure to antipsychotics was associated with a significantly increased risk of EPS compared with placebo (RR = 2.24, 95% CI = 1.71–2.93, p < 0.0001), corresponding to a number needed to harm of 7 (95% CI = 5–13). The highest risk estimates compared with placebo were observed for ziprasidone (RR = 3.59, 95% CI = 2.03–6.35, k = 3), risperidone (RR = 2.84, 95% CI = 1.89–4.28, k = 11), and aripiprazole (RR = 2.07, 95% CI = 1.12–3.82, k = 9). A higher proportion of female participants was associated with an increased risk of EPS (β = 0.0136, 95% CI = 0.0019–0.0254, p = 0.023), whereas longer treatment duration was associated with reduced risk (β=−0.0349, 95% CI = − 0.0573–−0.0124, p = 0.002). No significant associations were observed for diagnosis, age, ethnicity, antipsychotic exposure, or non-antipsychotic concomitant medication use (all p > 0.05).

**Conclusions:**

Antipsychotic drugs are associated with EPS in approximately 1 out of 7 C&A with a more than twofold increased risk compared with placebo. The highest risk estimates were observed for ziprasidone, risperidone, and aripiprazole. Careful risk–benefit assessment, tailored prescribing, and systematic monitoring of EPS in routine paediatric clinical practice are warranted.

## Background

Antipsychotic drugs have been increasingly prescribed to children and adolescents (C&A) over recent decades (1–7). Although originally developed for the treatment of schizophrenia in adults, the use of antipsychotics has expanded to a broad range of neuropsychiatric and neurodevelopmental conditions in paediatric populations, including autism spectrum disorder, bipolar disorder I, conduct disorder, and Tourette syndrome, among others (8, 9). This expansion has been accompanied by a substantial increase in off-label prescribing, often extrapolated from adult evidence (5, 10–12). Real-world data further indicate that fewer than half of C&A receiving antipsychotics have a recorded mental health diagnosis, with autism spectrum disorder representing the most common indication, and risk-benefit discussion rarely comprehensively documented (1, 2, 9).

Physical health monitoring and other relevant decision-making factors are frequently not explicit in clinical records (3, 5, 13). The use of antipsychotics in C&A has increasingly raised concerns regarding safety (7, 14, 15), particularly in relation to physical adverse effects (4, 7, 9, 11, 16, 17), which remain inconsistently reported as most clinical trials are primarily designed to assess efficacy (11).

Extrapyramidal symptoms (EPS)—including parkinsonism, akathisia, dystonia, and tardive dyskinesia (18, 19)—are among the most clinically relevant antipsychotic-related events in C&A (11). EPS can impair quality of life in C&A, disrupting academic functioning, limiting social participation, and contributing to stigma and poorer treatment adherence (20, 21). C&A may be particularly vulnerable to EPS due to ongoing neurodevelopmental processes, including synaptic pruning, progressive myelination, and maturation of fronto-striatal dopaminergic circuits, which are centrally involved in both psychiatric and neurological disorders (22, 23). Furthermore, neurodevelopmental conditions such as autism spectrum disorder may further increase susceptibility to EPS (1, 24). While EPS risk and prevalence are well-established in adults (25–28), evidence in paediatric population remains limited (6, 8, 15). The prevalence of antipsychotic-related EPS in adults has been meta-analytically estimated at approximately 31% (25), whereas clinical trials in paediatric samples indicate an increased risk of EPS with several antipsychotics, with reported relative effect estimates ranging from 3 to 26 compared with placebo (7, 14, 15, 29).

Previous evidence in paediatric population has largely focused on specific conditions—including a systematic review in autism spectrum disorder and an umbrella review in early onset psychosis (15, 24)—limiting the transdiagnostic synthesis of findings. A prior narrative review has described neurological adverse effects associated with antipsychotics across a wide range of mental health conditions in young people, but did not apply formal meta-analytic methods, thereby precluding quantitative estimation and comparison of EPS risk across disorders (12). To the best of our knowledge, no meta-analysis has systematically quantified the risk of antipsychotic-related EPS across diagnostic categories in C&A.

Given the increasing and transdiagnostic use of antipsychotics in C&A, a comprehensive quantitative synthesis of EPS risk across mental health conditions seems warranted. Accordingly, this study aims to address this gap by meta-analytically estimating the risk of EPS in C&A exposed to antipsychotics compared with placebo across a wide range of neuropsychiatric and neurodevelopmental disorders, and to explore potential sociodemographic, clinical, and pharmacological moderators.

## Methods

The protocol for this systematic review and meta-analysis was registered on PROSPERO (CRD42024564227), and the study is reported in accordance with the Preferred Reporting Items for Systematic Reviews and Meta-Analyses (PRISMA) guidelines (30).

### Literature search

A systematic literature search was performed in MEDLINE, EMBASE, and Web of Science from database inception until the 4th of July 2024, and updated on the 6th of January 2026. The combined search strategy applied across these three databases has demonstrated high sensitivity in validation exercises, retrieving approximately 96% of reference set of known eligible studies (31). No methodological filters restricting study design were applied beyond those inherent to the search strategy. Search terms combined controlled vocabulary and free-text terms related to antipsychotic drugs, C&A, and RCTs (full search strategy is provided in Supplementary Text: Search strategy).

Articles identified were screened at the title and abstract level, and after the exclusion of records not meeting the inclusion criteria, the full texts of the remaining articles were assessed for eligibility by three pairs of independent reviewers (JG&MAL; GD&IE; NC&LLL).

### Inclusion and exclusion criteria

Studies were eligible for inclusion if they met the following criteria: a) Study design: double-blind, parallel-group RCTs comparing antipsychotics with placebo. b) Population: C&A (age range < 18 years) receiving antipsychotics with no restrictions regarding diagnosis. c) Intervention and Comparator: Antipsychotics administered as monotherapy (i.e., no concomitant antipsychotic use), compared with placebo. d) Outcomes and reporting: studies reporting EPS, either as adverse events or assessed using validated rating scales, with sufficient data to extract or derive the number of participants experiencing EPS in both antipsychotic and placebo groups (including counts, proportions, or data allowing dichotomisation).

Studies were excluded in a hierarchical manner if they did not meet these criteria, including a) non-randomised or open label studies; b) non-parallel designs (e.g. crossover trials); c) compared antipsychotics only with active comparators without a placebo arm; d) evaluated antipsychotic polypharmacy. No language restrictions were applied.

When multiple publications reported results from the same trial, the most complete study—defined as the largest sample size or most comprehensive EPS data—was included in the meta-analysis to avoid double-counting of participants. In cases of complete overlap, the most recent publication was retained.

EPS were broadly defined as drug-induced movement disorders, including parkinsonism, akathisia, dystonia, and dyskinesia, consistent with established clinical classifications of antipsychotic-related movement disorders (detailed operational definitions and the specific assessment methods used are provided in Supplementary Text) (12, 18, 32).

### Data extraction strategy

Three pairs of researchers (JG&MAL; GD&IE; NC&LLL) independently extracted data from included studies into a database (Microsoft Excel). Discrepancies were resolved through discussion within each reviewer pair; when consensus could not be reached within the pair, a third reviewer (JG), consulting with the senior authors (GSP&CDC), helped reach a final decision. Extracted variables included: first author and year of publication; trial registration number (NCT); country; sample size (total, antipsychotic-treated group, and placebo group); antipsychotic used as monotherapy; mean daily dose expressed in chlorpromazine equivalents (CPZE, mg/day)—calculated using published antipsychotic dose equivalence estimates based on the minimum effective dose method (33)—; study duration (weeks); allowance of concomitant medication other than antipsychotics (yes vs. no); age (range and mean ± SD for antipsychotics and placebo groups); sex (% female); ethnicity (% “non-White”); diagnosis; EPS assessment; events of EPS reported (n and % in the antipsychotics and placebo groups) (Table 1).

EPS were operationalised as a single dichotomous outcome (presence vs absence), irrespective of subtype. Studies reporting EPS as adverse events or categorical outcomes were included directly. When EPS were assessed using validated clinical rating scales, data were extracted and dichotomised, when necessary, to indicate the presence or absence of EPS according to thresholds defined in the original studies. When multiple EPS measures or time points were reported, the endpoint corresponding to the longest follow-up during the double-blind treatment phase was prioritised. When more than one validated scale was used, the presence of EPS was determined according to the criteria specified by the original study; if necessary, a composite dichotomous outcome was derived, whereby EPS were considered present if any scale or endpoint indicate the presence of EPS according to study-defined criteria.

### Risk-of-bias assessment

The risk of bias in the included RCTs was independently assessed by two researchers (GD&IE) using the Cochrane Risk of Bias tool for randomised trials (RoB2) (34). Bias was evaluated across the five RoB2 domains: bias arising from the randomization process, bias due to deviations from intended interventions, bias due to missing outcome data, bias in measurement of the outcome, and bias in selection of the reported result. Each domain was rated as *low risk*, *some concerns*, or *high risk*, according to the RoB2 algorithm. Discrepancies between researchers were resolved by discussion, with consultation of a third researcher (JG) when needed. An overall risk-of-bias judgement was assigned to each trial based on the highest level of risk identified across domains.

### Data synthesis strategy

The primary quantitative synthesis estimated the relative risk (RR) of any EPS associated with antipsychotics versus placebo in C&A. Random-effects metaanalyses were conducted using risk ratios with 95% confidence intervals (CIs), pooled via the inverse-variance method. Between-study heterogeneity was estimated using the restricted maximum-likelihood (REML) estimator, and the Hartung–Knapp adjustment was applied to provide more conservative inference under random-effects assumptions (35). Statistical heterogeneity was assessed using Cochran’s Q test and quantified using τ^2^ and the I^2^, with 95% CIs for heterogeneity derived using the Q-profile method. Continuity correction of 0.5 was applied to studies with zero cell frequencies, while trials with zero events in both arms were retained, following standard recommendations for meta-analysis of sparse binary data (36).

As secondary outcomes, we estimated the number needed to harm (NNH). The NNH was calculated by applying the pooled RR to the pooled control event rate derived from the placebo arms, in accordance with established methods for translating relative effects into absolute measures of harm (37). 95% CI for the NNH were obtained by applying the same formula to the lower and upper bounds of the RR 95% CI (37). As an additional secondary outcome, we estimated the pooled prevalence of any EPS in antipsychotic-treated participants, synthesised using random-effects model with logit transformation of proportions.

Study-level meta-regressions were conducted to explore potential sources of heterogeneity in the primary outcome (RR of EPS). Separate mixed-effects meta-regression models were fitted for each moderator, including a) age (mean), b) sex (female %), c) ethnicity (“non-White” %), d) duration of treatment (weeks), and e) antipsychotic dose (CPZE mg/day). Moderator values were extracted at the study level and reflected the characteristics of the overall trial sample. Models were fitted using the log-transformed RR (log RR) as the dependent variable and estimated using restricted maximum likelihood (REML). Residual between-study heterogeneity was quantified using τ^2^, and the significance of moderator effects was assessed using Wald-type tests.

Prespecified subgroup analyses were conducted to explore heterogeneity in EPS risk by a) individual antipsychotic vs b) baseline diagnosis vs c) non-antipsychotic concomitant medication (yes vs no). Pooled estimates were calculated for RR using mixed-effects models with inverse-variance weighting, including continuity corrections for zero-event studies (36). For diagnosis-based subgroup analyses, only trials enrolling participants with a single primary diagnosis were considered, to avoid misclassification across diagnostic categories. Trials including mixed diagnostic populations were therefore excluded from this specific subgroup analysis. Between-study heterogeneity was quantified using τ^2^, I^2^, and Cochran’s Q, and subgroup differences were tested using a Q test for subgroup differences under a random-effects framework.

In line with Cochrane recommendations (36), meta-regression analyses were conducted only when at least 10 studies were available for a given moderator, while subgroup analyses were performed when a minimum of two studies contributed to each subgroup.

Additional sensitivity analyses were conducted after excluding a) studies rated as with high risk of bias according to RoB2, and b) studies that did not use validated EPS assessment scales. We re-ran the meta-analysis to assess the robustness of the primary results.

All quantitative syntheses were conducted in RStudio (version 2026.01.1 + 403) using the R packages *meta*, *metafor*, and *netmeta*. Statistical significance was set at p < 0.05 and assessed using two-tailed tests.

## Results

**Figure 1:**
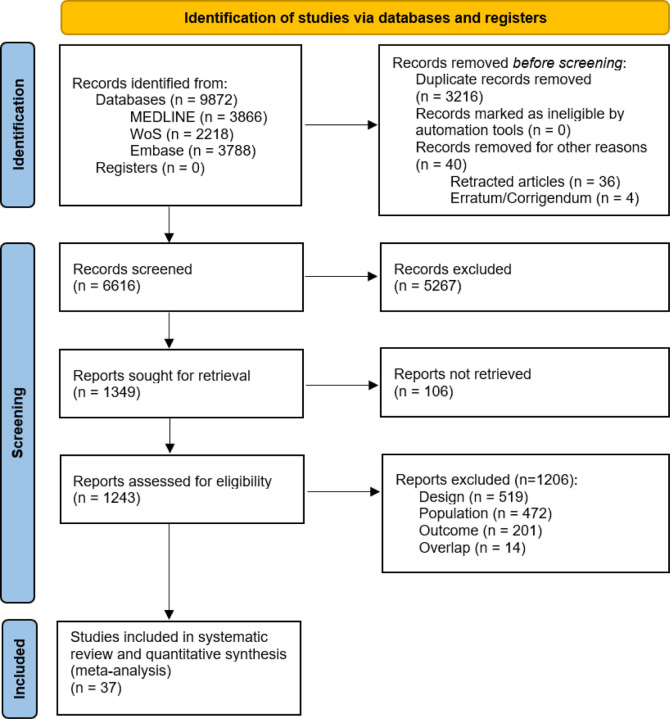
PRISMA flowchart 2020

We identified 9,872 records. After removal of 3,216 duplicates and exclusion of 40 records prior to screening due to retracted status (n = 36) or errata/corrigenda (n = 4), 6,616 records were screened by title and abstract, of which 5,267 were excluded. Full texts of 1,243 articles were assessed for eligibility (106 reports could not be retrieved), and 1,206 were excluded for reasons detailed in Fig. 1. Thirty-seven studies (38–74) were included in the systematic review and quantitative synthesis. Figure 1 shows the PRISMA flowchart of the study selection process.

### Study characteristics

The included trials comprised 5,128 participants (3,141 receiving antipsychotics and 1,987 receiving placebo). The mean age was 12.8 years (SD = 3.54; range 4–16.1) in the antipsychotic group and 12.4 years (SD = 3.62; range 4–16.3) in the placebo group. Females comprised 38.4% of the antipsychotic group and 32.2% of the placebo group. Participants of “non-White” ethnicity represented 26.9% in the antipsychotic group and 24.6% in the placebo group (Table 1).

A total of 481 EPS events were reported across the groups. The pooled prevalence of EPS in the antipsychotic group was 11.1% (95% CI = 7.8–15.5), with individual study estimates ranging from 0% to 42.9%. Reporting of specific EPS subtypes was heterogeneous across trials, precluding formal quantitative synthesis. Descriptively, parkinsonism and akathisia were the most frequently assessed and reported EPS domains across trials, whereas dystonia and dyskinesia were less consistently evaluated. Among studies reporting these outcomes, prevalence estimates varied widely across antipsychotic-treated groups, with dyskinesia ranging from 0% to 16%, parkinsonism from 0% to 15%, akathisia from 0% to 11%, and dystonia from 0% to 10%. When reported, these events were generally more frequent in antipsychotic-treated participants than in placebo groups. EPS were assessed using validated rating scales for antipsychotic-associated movement disorders in most studies (32), including the Abnormal Involuntary Movement Scale (AIMS), Barnes Akathisia Rating Scale (BARS), Drug-Induced Extrapyramidal Symptoms Scale (DIEPSS), Extrapyramidal Symptoms Rating Scale (ESRS), and the Simpson–Angus Scale (SAS); some trials reported EPS based on other clinical assessment methods (38, 49, 57, 58, 63, 70) (Table 1).

The antipsychotic drugs of study across trials included in this work were risperidone (k = 11), aripiprazole (k = 9), ziprasidone (k = 3), brexpiprazole (k = 2), lurasidone (k = 2), quetiapine (k = 2), asenapine (k = 1), blonanserin (k = 1), haloperidol (k = 1), molindone (k = 1), olanzapine (k = 3), and paliperidone (k = 1) (Fig. S2). Included trials enrolled participants with a range of baseline diagnoses, most commonly autism spectrum disorder (k = 12), schizophrenia (k = 11), bipolar disorder I (k = 5), Tourette syndrome (k = 3), and conduct disorder (k = 2) (Fig. S3).

### Risk-of-bias assessment

Of the thirty-seven included trials, 10 (27%) were judged to be at low risk of bias, 23 (62.2%) raised some concerns, and 4 (10.8%) were rated as high risk overall. Judgements of ‘some concerns’ were primarily driven by issues related to the randomisation process (D1) and reporting of prespecified outcomes (D3). Only a minority of studies (11%) (38, 40, 44, 61) were considered at high risk, suggesting that the overall evidence base was predominantly of low or moderate methodological concern (Fig. S1).

### Risk of extrapyramidal symptoms in antipsychotic-treated children and adolescents

**Figure 2:**
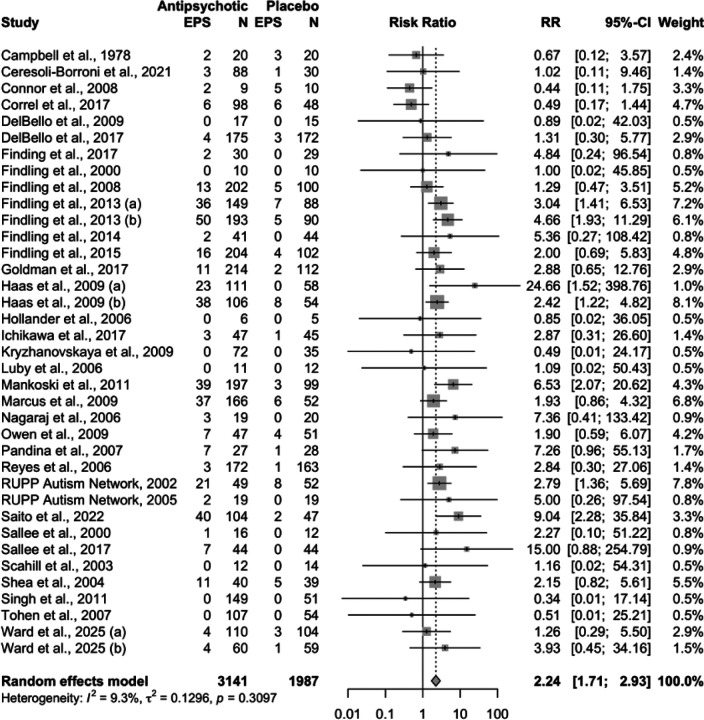
Forest plot Continuity correction of 0.5 was applied where required. EPS: extrapyramidal symptoms; RUPP: Research Units on Paediatric Psychopharmacology

Exposure to antipsychotics was associated with a significantly increased risk of EPS in C&A compared with the placebo group (RR = 2.24, 95% CI = 1.71–2.93, p < 0.0001) (Fig. 2).

Based on the pooled control event rate, the NNH was estimated at 7 (95% CI = 5–13).

### Risk of extrapyramidal symptoms in children and adolescents by antipsychotic

Thirty-three RCTs contributed to the subgroup meta-analysis by antipsychotic with significant differences in EPS risk across drugs observed (QM = 86.53, df = 12, p < 0.0001). The highest pooled RR was found for ziprasidone (RR = 3.59, 95% CI = 2.03–6.35, k = 3), risperidone (RR = 2.84, 95% CI = 1.89–4.28, k = 11), and aripiprazole (RR = 2.07, 95% CI = 1.12–3.82, k = 9) (in descending order of effect size). For other antipsychotics, effect estimates were not significantly different from placebo, including lurasidone (RR = 1.94, 95% CI = 0.68–5.54, k = 2), brexpiprazole (RR = 1.81, 95% CI = 0.54–6.11, k = 2), and quetiapine (RR = 0.44, 95% CI = 0.11–1.75, k = 2) (Fig. S2).

### Risk of extrapyramidal symptoms in children and adolescents by diagnosis

Twenty-four RCTs were included in the diagnosis-based subgroup meta-analyses, restricted to trials enrolling participants with a single primary diagnosis (i.e., excluding mixed diagnostic samples). There was no evidence of statistically significant differences between diagnostic subgroups (Q = 7.87, df = 4, p = 0.097), indicating that effect estimates did not differ reliably across diagnoses. Across individual diagnostic groups, point estimates suggested an increased risk of EPS compared with placebo in bipolar disorder I (RR = 3.25, 95% CI = 1.81–5.84, k = 3), autism spectrum disorder (RR = 2.26, 95% CI = 1.52–3.36; k = 10), and schizophrenia (RR = 2.13, 95% CI = 1.23–3.66, k = 8) (in descending order of effect sizes), whereas estimates for Tourette syndrome were imprecise and not statistically significant from placebo (RR = 6.39, 95% CI = 0.79–51.92, k = 2). Estimates for conduct disorder were not reported due to insufficient data (k = 1) (Fig. S3).

### Risk of extrapyramidal symptoms in children and adolescents by concomitant medication

Thirty-seven RCTs contributed to the subgroup analyses stratified by allowance of concomitant medication (other than antipsychotics). There was no evidence of statistically significant differences between subgroups (Q = 1.71, df = 1, p = 0.192). Point estimates indicated an increased risk of EPS compared with placebo both in trials allowing concomitant medication (RR = 2.49, 95% CI = 1.95–3.18, k = 25) and in those not allowing concomitant medication (RR = 1.60, 95% CI = 0.79–3.23; k = 12), although the latter was imprecise and did not reach statistical significance (Fig. S4).

### Meta-regression analyses of study-level moderators of extrapyramidal symptoms risk

Study-level meta-regression identified two significant moderators of EPS risk. A higher percentage of female participants was associated with greater EPS risk (β = 0.0136, 95% CI = 0.0019–0.0254, p = 0.023). In contrast, longer treatment duration was associated with lower EPS risk (β=−0.0349, 95% CI = −0.0573–−0.0124, p = 0.002). No significant moderation effects were observed for age, ethnicity, antipsychotic dose (CPZE) (all p > 0.05) (Table 2).

### Sensitivity analyses

In sensitivity analyses excluding the four RCTs rated as high risk of bias (38, 40, 44, 61), the association between antipsychotic use and EPS risk remained statistically significant and of similar magnitude (RR = 2.48, 95% CI = 1.91–3.22, p < 0.0001).

In sensitivity analyses excluding the six studies that did not use validated EPS assessment scales (38, 49, 57, 58, 63, 70), the association between antipsychotic use and EPS risk remained statistically significant and of similar magnitude (RR = 2.21, 95% CI = 1.62–3.02, p < 0.0001).

### Between-study heterogeneity

Overall, between-study heterogeneity was generally low to modest across analyses.

For the primary outcome of EPS risk (RR), heterogeneity was low (τ^2^=0.13; I^2^=9.3%) (Fig. 2), with Cochran’s Q test indicating no statistically significant heterogeneity. Subgroup analyses by antipsychotic showed negligible heterogeneity, while diagnosis-based subgroups ranged from negligible (I^2^=0% for autism spectrum disorder and Tourette syndrome) to moderate (I^2^=30–57% for bipolar disorder I and schizophrenia). For non-antipsychotic concomitant medication, heterogeneity was negligible in trials allowing concomitant treatment (I^2^=0%) and moderate in those without (I^2^=45.8%). Residual heterogeneity in meta-regression models was negligible, and sensitivity analyses excluding high-risk-of-bias trials or those without validated EPS scales demonstrated similarly low heterogeneity (I^2^=0–12%) (Table 2).

## Discussion

To the best of our knowledge, this comprehensive meta-analysis of double-blind RCTs provides the first quantitative estimate of EPS risk associated with antipsychotics in C&A across diagnostic categories. In relative terms, antipsychotic exposure was associated with a more than twofold increase in EPS risk compared with placebo, corresponding to a number needed to harm of 7. In sensitivity analyses, we found similar effect sizes that remained statistically significant indicating that the results are robust, and the overall low heterogeneity across analyses shows that the estimated effects were generally consistent across studies. Previous meta-analyses of RCTs in schizophrenia (75) have reported relative effects (1.61–4.76) and NNH values (3–11) broadly consistent with our findings. Thus, the relative EPS risk associated with antipsychotics in C&A appears comparable to that observed in adults under controlled conditions. Notably, prior studies have suggested that C&A may exhibit increased sensitivity to antipsychotic-induced EPS compared with adults (6, 12, 14, 29).

EPS risk varied significantly across antipsychotics, indicating meaningful differences in tolerability; however, interpretation should be cautious given the limited number of trials for several drugs. Across RR analyses, the most consistent signals were observed for ziprasidone, risperidone, and aripiprazole, which were associated with increased EPS risk compared with placebo and supported by the largest evidence base. These findings are broadly consistent with previous evidence reporting higher EPS risk with ziprasidone, risperidone, and aripiprazole in paediatric populations (6, 11, 12, 14, 29, 76–79). In contrast, other drugs did not show significant differences from placebo, although these estimates were generally based on few studies and were therefore more imprecise. Lower-intermediate risk estimates were observed for brexpiprazole, quetiapine, and lurasidone, with a small number of available trials. Quetiapine has showed a favourable EPS profile in prior literature (6, 14, 29), although other paediatric studies have reported higher rates of EPS with quetiapine relative to placebo (12, 79). Lurasidone has previously been associated with EPS in C&A (11, 15), whereas evidence remains limited for brexpiprazole. Other studies have identified an association between olanzapine and EPS in paediatric populations (11, 79), while additional evidence suggests a lower risk of EPS with olanzapine compared with risperidone (15); however, in the two trials included in our review, no EPS events were reported in either arm. Clozapine was not represented as no RCT met the inclusion criteria for the present study; nevertheless, previous reports have described low EPS rates in paediatric samples (11, 12, 29). Taken together, these findings support the view that antipsychotic-related EPS risk in C&A is not uniform across drugs. The negligible between-study heterogeneity observed within agent-specific analyses suggests internally consistent trial-level estimates; nonetheless, the strength of inference for several compounds remains constrained by the limited paediatric evidence base.

We found no evidence of statistically significant differences in EPS risk across diagnostic subgroups. Nevertheless, effect estimates consistently suggested an increased risk across bipolar disorder I, autism spectrum disorder, and schizophrenia, with the most precise estimates observed in autism spectrum disorder, which contributed the largest number of studies. Given that autism spectrum disorder represents one of the most common indications for antipsychotic prescribing in C&A (9), these findings may be of particular clinical relevance. Prior evidence suggests heightened vulnerability to antipsychotic-induced EPS in neurodevelopmental conditions (1, 24), potentially related to alterations in dopaminergic signalling, fronto-striatal circuitry, and baseline motor functioning (1, 24). Overall, diagnostic category alone does not appear to account for variability in EPS risk, and the absence of statistically significant between-group differences, together with the limited number of studies and substantial heterogeneity in some subgroups, suggests that these findings should be interpreted cautiously and considered exploratory.

Meta-regression analyses identified the proportion of female participants at study level as a significant moderator of EPS risk, with higher female representation associated with higher RR. This finding is consistent with prior reports suggesting higher rates of EPS in females than males treated with antipsychotics in C&A (6, 29). Biological mechanisms may plausibly contribute to this pattern, including sex-related genetic variations affecting dopaminergic signalling and hormonal modulation of dopamine receptor sensitivity, both of which may influence vulnerability to EPS (80, 81). The inverse association between treatment duration and EPS risk may reflect early onset of acute EPS followed by partial attenuation over time, selective discontinuation of more susceptible individuals, or dose optimisation during longer trials. For comparative purposes, longitudinal data in adults remain mixed: some studies have reported a decrease (82), whereas others an increase (83) in overall EPS rates after one year of antipsychotic exposure, with variability also observed across EPS subtypes (84). CPZE was not identified as a significant moderator, despite prior meta-analytic evidence in adult populations supporting a dose-response relationship for EPS (85). This discrepancy may reflect the relatively narrow dosing ranges in paediatric trials or the inherent limitations of study-level meta-regression. As these meta-regression analyses were based on study-level covariates extracted from each trial, they are subject to ecological bias and should therefore be interpreted with caution and considered hypothesis-generating rather than confirmatory.

### Clinical implications

EPS carry important clinical implications, informing both risk-benefit decision-making in prescribing and the understanding of illness course. In C&A, EPS may disrupt compliance and functioning, contributing to stigma (12, 20, 21, 29) and adversely affecting overall mental and physical health. EPS have been associated with poorer outcomes—including cognitive impairment, educational problems, and lower academic attainment— in first-episode psychosis, even in antipsychotic-naïve individuals, suggesting that they may reflect underlying neurobiological vulnerability rather than solely agent-related effects (83, 86–88). EPS may thus provide clinical signals of altered dopaminergic function and ongoing neurodevelopmental processes involving both psychiatric and neurological symptoms (22, 23, 89). Further research is warranted to clarify the prognostic significance of EPS across diagnostic groups beyond schizophrenia.

These considerations are particularly relevant given the increasing and often off-label use of antipsychotics in C&A, frequently in settings where evidence for efficacy and safety remains limited (5, 9, 29). Clinical guidance (NICE) recommends a balanced risk-benefit decision-making in prescribing antipsychotics, contrasting side-effect profiles of different drugs (90, 91) to reduce the adverse-effect burden in C&A—many of whom continue treatment into adulthood— and yield both short- and long-term health benefits (4). However, real-world data suggest that physical health monitoring among C&A receiving antipsychotics remains suboptimal (9). Strengthening adherence to monitoring standards and streamlining guidance may support safer prescribing across care settings (9). A more comprehensive assessment of benefits—including quality of life, severity of maladaptive behaviours, residential and educational placement, and behaviour-related health issues—along with detailed characterization of sample features (e.g., intellectual and adaptive functioning, autonomy), should be systematically recorded to enable appropriate risk–benefit decision-making.

Standardised monitoring of EPS in clinical practice may be implemented through validated rating scales (32, 89) combined with routine neurological examination (89). Instrumental assessment, including actigraphy and other emerging wearable digital approaches, enables long-term real-world monitoring and provides a more objective, sensitive, and ecological assessment of subthreshold psychomotor behaviours (19, 89).

These implications align with current consensus statements addressing the importance of systematic neurological assessment within psychiatric care, particularly in paediatric populations (89). Enhancing neuropsychiatric training among clinicians working with C&A may improve early detection of EPS and optimise risk-benefit decision-making in the context of rising antipsychotic prescribing, contributing to a more integrated and holistic model of care (89).

### Limitations and strengths

Several limitations should be acknowledged. First, although statistical heterogeneity was low, clinical variability across trials—diagnoses, antipsychotic drugs, dosing strategies, and treatment duration—may limit direct generalisability to specific patient subgroups. Second, most included trials were of relatively short duration, restricting conclusions regarding long-term EPS risk. Third, the limited number of studies for certain antipsychotics, such as first-generation antipsychotics, resulted in imprecise estimates with wide confidence intervals, warranting cautious interpretation of drug-specific comparisons. Fourth, although outcomes were harmonised into a dichotomous presence/absence EPS variable (86), differences in measurement instruments and reporting thresholds across trials may have introduced residual heterogeneity and precluded separate quantitative synthesis of specific EPS subtypes; therefore, these outcomes were summarised descriptively. Fifth, none of the included RCTs used instrumental assessment, potentially reducing sensitivity and leading to underestimation or misclassification of EPS. Future studies in C&A should incorporate instrumental-based measures to better characterise psychomotor behaviour (19, 89). Finally, although prespecified subgroup and meta-regression analyses were conducted, these should be regarded as exploratory, particularly where the number of contributing studies was small.

Nonetheless, the inclusion of a large, pooled sample of RCTs enhances the precision of the overall effect estimate. The use of double-blind placebo-controlled trials enhances comparability across studies. Results remained consistent in sensitivity analyses, supporting the internal validity of the findings. Absolute risk estimates, including the number needed to harm, are provided alongside relative measures to facilitate direct clinical interpretation. The analysis of EPS risk across diagnostic groups further supports the applicability of the findings in routine paediatric practice.

## Conclusions

Antipsychotics drugs are associated with a more than twofold increased risk of extrapyramidal symptoms compared with placebo in children and adolescents, with relative effect sizes broadly comparable to those observed in adults, and a meaningful variability across drugs. These findings highlight the importance of careful risk-benefit assessment, targeted prescribing, and systematic monitoring in paediatric populations. Future research should prioritise developmentally appropriate evaluation and more consistent reporting of adverse events and psychomotor outcomes, including long-term longitudinal assessments, to better characterise the risk of extrapyramidal symptoms associated with antipsychotics in children and adolescents.

## Supplementary Material

Supplementary Files

This is a list of supplementary files associated with this preprint. Click to download.

- suppmaepsca.docx

## Figures and Tables

**TABLE 1:** Demographic and clinical characteristics

Study	Country	N *	Age: mean (SD) *	% female *	% “non-White” *	Diagnosis	Antipsychotic	CPZE	Weeks	% EPS *
Campbell et al., 1978 ^46^	USA	20 / 20	4.5 (NA) / 4.5 (NA)	NA	NA	ASD	Haloperidol	89.6	8	10% / 15% ^6^
Ceresoli-Borroni et al., 2021 ^47^	USA	88 / 30	9 (1.8) / 9 (1.7)	12.5% / 13.3%	35.2% / 40%	Mixed	Molindone^†^	150	6.5	3.4% / 3.3% ^1,2,3^
Connor et al., 2008 ^48^	USA	9 / 10	13.1 (1.2) / 15 (1.4)	22% / 30%	22% / 30%	CD	Quetiapine	207	7	22.2% / 50% ^1,2,3^
Correl et al., 2017 ^49^	Multi	98 / 48	15.3 (1.2) / 15.5 (1.1)	36.7% / 29.2%	33.7% / 45.8%	SZ	Aripiprazole	299	52	6.1 / 12.5% ^1,2,3^
DelBello et al., 2009 ^50^	USA	17 / 15	16 (2) / 15 (2)	71% / 67%	NA	BD I	Quetiapine^†^	283.8	8	0% / 0% ^1,2,3^
DelBello et al., 2017 ^51^	Multi	175 / 172	14.2 (2.2) / 14.3 (2.3)	49.1% / 48.8%	22.5% / 26.5%	BD I	Lurasidone^†^	210	6	2.2% / 1.74% ^1,2,3^
Findling et al., 2000 ^52^	USA	10 / 10	10.7 (3.4) / 8.2 (1.9)	NA	NA	CD	Risperidone	95.4	10	0% / 0% ^1,3^
Findling et al., 2008 ^53^	Multi	202 / 100	15.5 (1.3) / 15.4 (1.4)	45.5% / 39%	42.5% / 36%	SZ	Aripiprazole^†^	301	6	6.4% / 5% ^2,3^
Findling et al., 2013 (a) ^54^	USA	149 / 88	13.6 (NA) / 13.7 (NA)	43.6% / 46.6%	18.8% / 18.1%	BD I	Ziprasidone^†^	186.1	4	24.1% / 7.9% ^1,2,3^
Findling et al., 2013 (b) ^55^	Multi	193 / 90	15.3 (NA) / 15.4 (NA)	43.4% / 31.1%	15.5% / 33.3%	SZ	Ziprasidone^†^	195.1	6	25.9% / 5.5% ^1,2,3^
Findling et al., 2014 ^56^	USA	41 / 44	10.1 (2,8) / 10.8 (2.8)	26.8% / 13.6%	24.3% / 36.4%	ASD	Aripiprazole^†^	132.4	16	4.8% / 0% ^1,2,3^
Findling et al., 2015 ^57^	Multi	204 / 102	15.3 (1,5) / 15.4 (1.4)	36.7% / 39.2%	46.5% / 44.1%	SZ	Asenapine^†^	131.2	8	7.8% / 3.9% ^7^
Finding et al., 2017 ^58^	USA	30 / 29	11.7 (2.4) / 11.8 (3)	33.3% / 5.2%	NA	Mixed	Aripiprazole^†^	112.9	12	6.6% / 0% ^2,3^
Goldman et al., 2017 ^59^	Multi	214 / 112	15.4 (1.3) / 15.3 (1.4)	36% / 36.7%	31.6 / 34%	SZ	Lurasidone^†^	156	6	5.1% / 1.7% ^1,2,3^
Haas et al., 2009 (a) ^60^	USA	111 / 58	NA	51.3% / 52%	48% / 22%	Mixed	Risperidone^†^	NA	3	20.7% / 0% ^1,2,3^
Haas et al., 2009 (b) ^61^	Multi	106 / 54	15.7 (1.3) / 15.5 / (1.4)	72% / 35%	46.2% / 50%	SZ	Risperidone^†^	NA	6	35.8% / 14.8% ^1,2,3^
Hollander et al., 2006 ^62^	USA	6 / 5	9.2 (2.9) / 8.9 (2.1)	0% / 40%	50% / 20%	ASD	Olanzapine	210	8	0% / 0% ^3^
Ichikawa et al., 2017 ^63^	Japan	47 / 45	10.3 (3.3) / 9.9 (3.1)	17% / 20%	NA	ASD	Aripiprazole^†^	88.7	8	6.3% / 2,2% ^2,3,4^
Kryzhanovskaya et al., 2009 ^64^	Multi	72 / 35	16.1 (1.3) / 16.3 (1.6)	29.2% / 31.4%	27.8% / 35.6%	SZ	Olanzapine	233.6	6	0% / 0% ^1,2,3^
Luby et al., 2006 ^65^	USA	11 / 12	4 (0.9) / 4 (1.1)	18.1% / 33.3%	9% / 8%	ASD	Risperidone^†^	86.3	24	0% / 0% ^7^
Mankoski et al., 2011 ^66^	USA	197 / 99	13.6 (2.2) / 13.3 (2.1)	47.7% / 43.4%	32.4% / 39.4%	BD I	Aripiprazole	311.5	4	19.7% / 3% ^7^
Marcus et al., 2009 ^67^	USA	166 / 52	9 (2.8) / 10.2 (3.1)	11.4% / 7.7%	27.7% / 32.7%	ASD	Aripiprazole^†^	155.7	8	22.2% / 11.5% ^1,2,3^
Nagaraj et al., 2006 ^68^	India	19 / 20	4.8 (1.7) / 5.2 (1.6)	15.8% / 10%	NA	ASD	Risperidone^†^	72.7	24	15.7% / 0% ^3^
Owen et al., 2009 ^69^	USA	47 / 51	9.7 (3.2) / 8.8 (2.6)	10.6% / 13.7%	31.9% / 19.6%	ASD	Aripiprazole^†^	139.4	8	14.8% / 7.8% ^1,2,3^
Pandina et al., 2007 ^70^	Canada	27 / 28	7.4 (2.4) / 7.1 (2.1)	29.6% / 14.3%	40.7% / 35.7%	ASD	Risperidone	104	8	25.9% / 3.5% ^5^
Reyes et al., 2006 ^71^	USA	172 / 163	10.9 (2.9) / 10.8 (2.9)	18% / 9%	NA	Mixed	Risperidone^†^	75.7	36	1.7% / 0.6% ^7^
RUPP Autism Network, 2002 ^72^	USA	49 / 52	8.8 (2.7) / 8.8 (2.7)	20% / 17%	NA	ASD	Risperidone	136.3	8	42.8% / 15.3% ^1,2,3^
RUPP Autism Network, 2005 ^73^	USA	19 / 19	NA	NA	NA	ASD	Risperidone^†^	151.5	8	10.5% / 0% ^1,3^
Saito et al., 2022 ^74^	Japan	104 / 47	15.5 (1.5) / 15.6 (1.6)	57.7% / 47.4%	NA	SZ	Blonanserin^†^	292.5	6	38.4% / 4.2% ^4^
Sallee et al., 2000 ^75^	USA	16 / 12	11.3 (NA) / 11.8 (NA)	12.5% / 33.3%	NA	Tourette	Ziprasidone	56	8	6.2% / 0% ^1,3^
Sallee et al., 2017 ^76^	Multi	44 / 44	11.3 (2.9) / 11.6 (2.8)	41% / 25%	27.2% / 11.4%	Tourette	Aripiprazole^†^	156	8	15.9% / 0% ^1,2,3^
Scahill et al., 2003 ^77^	USA	12 / 14	11.1 (2.2) / 11.1 (2.2)	3.8% / 3.8%	NA	Tourette	Risperidone	189.4	8	0% / 0% ^3^
Shea et al., 2004 ^78^	Canada	40 / 39	7.6 (2.3) / 7.3 (2.3)	27.5% / 17.9%	32.5% / 28.2%	Mixed	Risperidone^†^	88.6	8	27.5% / 12.8% ^7^
Singh et al., 2011 ^79^	USA	149 / 51	15.3 (1.6) / 15.7 (1.4)	37% / 55%	32% / 31%	SZ	Paliperidone^†^	116.6	6	0% / 0% ^1,2,3^
Tohen et al., 2007 ^80^	USA	107 / 54	15.1 (1.3) / 15.4 (1.2)	NA	33.6% / 24%	BD I	Olanzapine^†^	187.3	3	0% / 0% ^1,2,3^
Ward et al., 2025 (a) ^81^	Multi	110 / 104	15.3 (1.5) / 15.3 (1.4)	53% / 56%	64% / 65%	SZ	Brexpiprazole^†^	300	6	3.6% / 2.8% ^1,2,3^
Ward et al., 2025 (b) ^82^	USA	60 / 59	9.8 (2.9) / 10 (3.2)	10% / 15.3%	81.7% / 76.3%	ASD	Brexpiprazole	168	8	6.6% / 1.7% ^1,2,3^

* Columns represent the two comparison arms (Antipsychotic / Placebo).

† Concomitant non-antipsychotic medication permitted during the trial.

1 SAS: Simpson–Angus Scale;

2 BARS: Barnes Akathisia Rating Scale;

3 AIMS: Abnormal Involuntary Movement Scale;

4 DIEPSS: Drug-Induced Extrapyramidal Symptoms Scale;

5 ESRS, Extrapyramidal Symptoms Rating Scale;

6 DOTES: Dosage Record and Treatment-Emergent Symptom Scale;

7 Other methods of EPS assessment (no validated scale).

ASD: autism spectrum disorder; BD: bipolar disorder; CD: conduct disorder; CPZE: chlorpromazine equivalents (mg/day); EPS: extrapyramidal symptoms; Multi: multicountry; N: sample size; NA: not applicable; NZ: New Zealand; RUPP: Research Units on Paediatric Psychopharmacology; SD: standard deviation; SZ: schizophrenia; USA: United States of America.

**Table 2:** Meta-regression analyses of study-level moderators

Moderator	Coefficient	95% CI	p
Age (years)	−0.0091	−0.0622 – 0.0805	0.802
**Female (%)**	0.0136	−0.0019 – 0.0254	**0.023**
“Non-White” (%)	−0.0020	−0.0245 – 0.0205	0.861
**Duration (weeks)**	−0.0349	−0.0573 - −0.0124	**0.002**
CPZE (mg/d)	0.0001	−0.0032 – 0.0033	0.961

(τ^2^ = 0, I^2^ = 0%). CPZE: chlorpromazine equivalents.

## Data Availability

The dataset generated for meta-analytic purposed were extracted from previously published trials and is fully reported in this published article, its tables and figures, and its supplementary information files.

## Abbreviations

C&A: Children and Adolescents
CI: Confidence Interval
CPZE: Chlorpromazine Equivalents
EPS: Extrapyramidal Symptoms
NICE: National Institute for Health and Care Excellence
NNH: Number Needed to Harm
PRISMA: Preferred Reporting Items for Systematic Reviews and Meta—Analyses
RCT: Randomised Placebo—Controlled Trial
REML: Restricted Maximum Likelihood
RR: Risk Ratio
SD: Standard Deviation
τ^2^: Between—Study Variance
I^2^: Heterogeneity Index
